# Sebaceous Lymphadenoma of Parotid: Imaging, Cytological, and Histological Findings in Detail

**DOI:** 10.1155/2018/2915907

**Published:** 2018-04-18

**Authors:** Golsa Shekarkhar, Hossein Soleimanpour, Seyed Hamed Jafari, Fatemeh Zamani

**Affiliations:** ^1^Pathology Department, Shiraz University of Medical Sciences, Shiraz 7134845794, Iran; ^2^Daneshbod Laboratory, Shiraz, Iran; ^3^Shiraz University of Medical Sciences, Shiraz, Iran

## Abstract

Sebaceous lymphadenomas are rare and account for less than 1% of primary salivary gland tumors. These rare tumors are mostly found in men older than 50 years. The clinicopathological features of these tumors are poorly understood and no definite causative factor has been reported for them till now. They are not often diagnosed prior to surgery, which could be due to their rarity and lack of enough preop radiological and cytological findings. Few case reports have been published in literature about their pathogenesis and accompanying malignant lesions. The cytological and imaging findings have been dealt with in some articles. Here we describe the histologic, cytologic, and radiologic findings of sebaceous lymphadenomas of parotid gland, all together, and discuss their differential diagnoses in various diagnostic methods.

## 1. Introduction

Sebaceous type cells are often found within normal parotid gland. Although they are found in a variety of lesions, primary neoplastic lesions with predominant sebaceous component, such as sebaceous adenoma and sebaceous lymphadenoma, are very rare and account for less than 1% of salivary gland tumors [1]. Here we describe the histological, cytological, and radiological findings of sebaceous lymphadenoma of parotid in a 39-year-old patient with an indolent clinical course and no other accompanying finding.

## 2. Case Presentation

A 39-year-old man was referred to ENT service with 4-month duration of left-sided, painless swelling in the face. Clinical examination revealed a semimobile, nontender, round mass in left parotid gland.

Sonographic findings revealed a hypoechoic mass measuring about 20 × 18 mm in left parotid gland suggestive of tumoral lesion. Multiple lymph nodes with benign appearance were also identified in left side of the neck.

**Figure 1:**
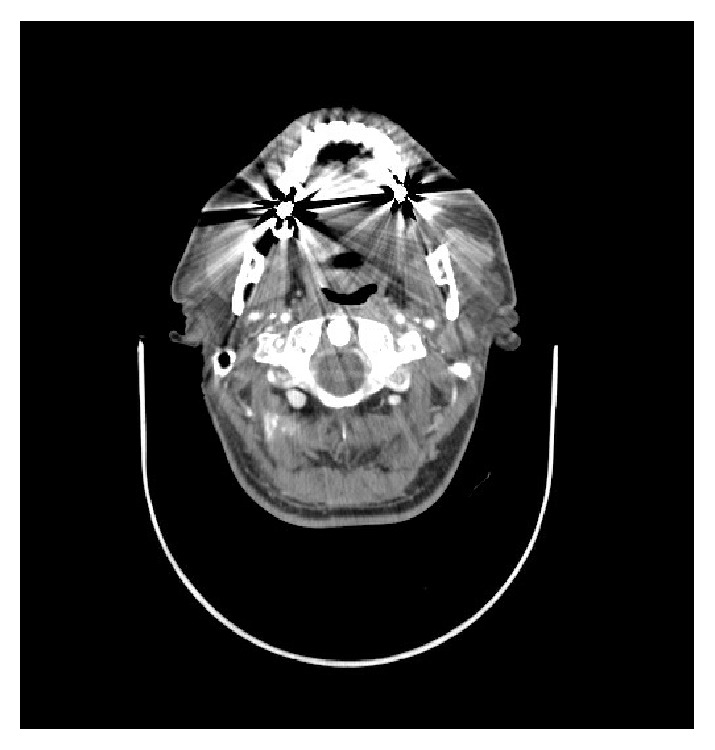
CT scan with contrast axial view. Despite severe metal streak artefacts due to presence of amalgam, a homogenous enhancing lesion measuring about 17*∗*15 mm is demonstrated in the deep portion of left parotid gland.

Spiral CT scan of head and neck with contrast showed a hyperdense soft tissue lesion measuring 17 × 15 mm in posteromedial part of left parotid gland suggestive of lymph node (Figure 1).

**Figure 2:**
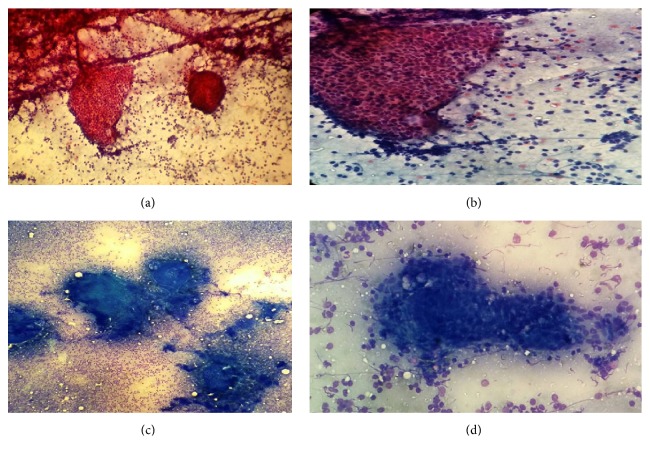
(a) & (b) low and high power view of aspirated material showing mixture of variably sized lymphocytes with few clusters of ductal epithelial cells ((a) ×10 and (b) ×40 papanicolaou stain). (c) & (d) low and high power view of the same material showing lymphocytic background with some dense proteinaceous material and ductal epithelial clusters ((a) ×10 and (b) ×40 wright stain).

The patient was scheduled for sonography guided-fine needle aspiration (FNA) from the mass. Prepared slides showed cellular smears, composed of numerous lymphocytes with variable size. Some clusters of acini and ductal epithelial cells were also present. There were no oncocytes or fat cells in prepared smears. Therefore the diagnosis was made as benign process, suggestive of intrasalivary gland lymph node with reactive changes (Figure 2).

**Figure 3:**
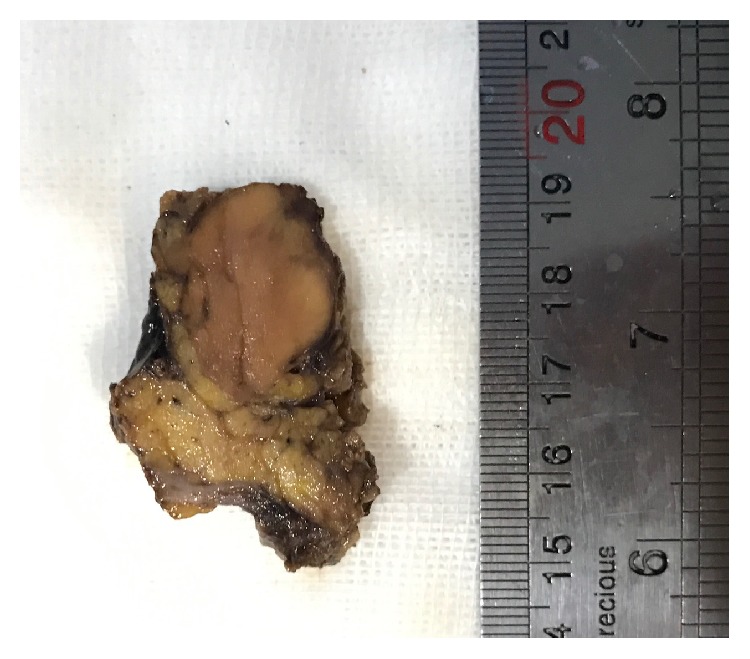
Gross appearance of mass. There is a well-defined tan-brown mass with solid cut surface attached to normal parotid tissue.

Excision of the mass was planned in a complete parotidectomy and the specimen was sent for pathology. Grossly there was a well-defined shiny light brown mass *M*: 3.5*∗*3*∗*2 cm with attached normal parotid gland tissue. No necrosis or hemorrhage was seen (Figure 3).

**Figure 4:**
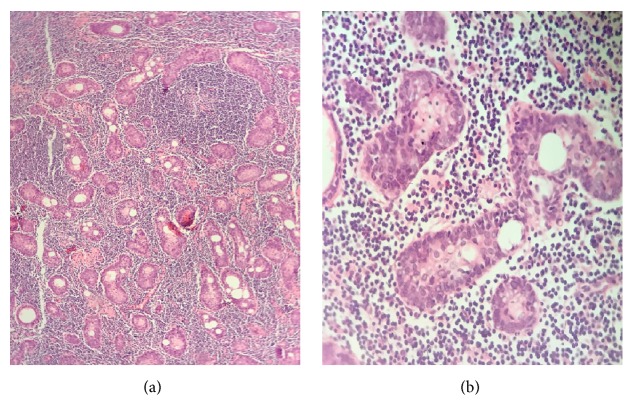
Sebaceous lymphadenoma micrograph ((a) ×10). The tumor is biphasic composed of epithelial nests and lymphoid background; ((b) ×40) solid nests with squamous and sebaceous differentiation.

Microscopically, the lesion was completely encapsulated and showed ductal epithelial components with squamous and sebaceous differentiation. The lymphoid background was composed of multiple lymphoid follicles with germinal centers (Figure 4). There was no evidence of residual lymph node architecture in the specimen. There was no giant cell reaction in the specimen.

The last follow-up of the patient was after 6 months and showed no sign of recurrence. The accompanying enlarged neck lymph nodes were also vanished.

## 3. Discussion

Sebaceous lymphadenoma is a rare, benign tumor with nests and islands of bland epithelium composed of sebaceous and squamous elements, in a prominent lymphoid stroma [2]. Over 90% occur in or near the parotid gland and the rest in minor salivary glands. It is not usually diagnosed prior to excision [2], as in our case, in which both imaging studies and FNA cytology could not indicate the correct diagnosis. The lesion shows a slight male preponderance as in other salivary gland tumors [2, 3]. This is similar to other sebaceous salivary gland tumors. This may be due to the generally higher concentration of sebaceous glands in men than in women.

The pathogenesis of the lesion and its relation to viruses has been discussed previously by Seethala et al. [3], and no evidence of EBV, HPV, or HHV-8 has been found. The accompanying malignant lesions reported in literature are acinic cell carcinoma and squamous cell carcinoma [4]. Malignant transformation of sebaceous lymphadenoma into sebaceous lymphadenocarcinoma is a very rare occurrence [4].

In imaging studies the contrast mediated CT scan was restricted by multiple metallic artefacts due to amalgam dental fillings. However it demonstrated a well-defined enhancing solid lesion in the deep portion of left parotid gland with Hounsfield unit 65. These imaging findings are not specific. Well-defined margin is characteristic in benign lesions but degree of contrast enhancement of tumor cannot distinct between benign and malignant [5, 6]. The most likely diagnosis with these findings is pleomorphic adenoma [5]. Majeed et al. reported a patient who had sebaceous lymphadenoma of parotid gland which was well circumscribed but appeared to be multiloculated cystic in MRI and made them think it is a Warthin tumor [7]. In contrast, in our patient, a well-defined totally solid lesion was identified. Therefore the differential diagnosis of Warthin was not considered for our patient.

In FNAC, the lesion is mostly diagnosed as intraparotid lymph node or Warthin tumor due to presence of numerous lymphocytes, bland looking ductal epithelial cells, and few oncocytes [3]. The diagnosis of Warthin was ruled out in our case due to absence of oncocytic cells. It should be noted that sebaceous component, otherwise prominent, usually cannot be detected in aspirated material.

FNAC can also be helpful as a preop diagnostic method, because it establishes that the lesion is either benign or nonneoplastic, or at least low grade.

In histology, by adequate sectioning, sebaceous component can be easily diagnosed. Lymphoid follicles with germinal centers help to differentiate this tumor from lymphoid neoplasms. Also the fact that this is a well circumscribed lesion helps us distinguish it from lymphoepithelial sialadenitis. Lack of mitosis or cellular atypia in the epithelial component distinguishes them from malignant counterparts.

In summary, in order not to miss it, one should keep in mind the diagnosis of sebaceous lymphadenoma in list of differential diagnoses when facing a lymphoepithelial lesion.
